# Effect of Walking Speed on the Reliability of a Smartphone-Based Markerless Gait Analysis System

**DOI:** 10.3390/s25206474

**Published:** 2025-10-20

**Authors:** Edilson Fernando de Borba, Jorge L. Storniolo, Serena Cerfoglio, Paolo Capodaglio, Veronica Cimolin, Leonardo A. Peyré-Tartaruga, Marcus P. Tartaruga, Paolo Cavallari

**Affiliations:** 1 Physical Education Department, Federal University of Paraná, Curitiba 81530-000, PR, Brazil; edilson.borba@ufpr.br (E.F.d.B.); mtartaruga@unicentro.br (M.P.T.); 2 Laboratorio Sperimentale di Fisiopatologia Neuromotoria, Istituto Auxologico Italiano, 20821 Meda, Italy; paolo.cavallari@unimi.it; 3 Department of Electronics, Information and Bioengineering, Politecnico di Milano, 20133 Milan, Italy; serena.cerfoglio@polimi.it (S.C.); veronica.cimolin@polimi.it (V.C.); 4 Research Laboratory in Biomechanics, Rehabilitation and Ergonomics, Istituto Auxologico Italiano, 28824 Piancavallo, Verbania, Italy; p.capodaglio@auxologico.it; 5 Department of Biomedical, Surgical and Dental Sciences, La Statale University, 20133 Milan, Italy; 6 Human Locomotion Laboratory (LOCOLAB), Department of Public Health, Experimental and Forensic Medicine, University of Pavia, 27100 Pavia, Italy; leonardoalexandre.peyretartaruga@unipv.it; 7 Physical Education Department, Midwestern Parana State University-UNICENTRO, Guarapuava 85040-080, PR, Brazil; 8 Human Physiology Section of the Department of Pathophysiology and Transplantation, Università Degli Studi, 20133 Milan, Italy

**Keywords:** gait analysis, OpenCap, MoCap, walking, biomechanics

## Abstract

**Highlights:**

This study tested OpenCap, a smartphone app that tracks movement without markers, by comparing it to a traditional setup at different walking speeds. OpenCap was really reliable for measuring movement timing and joint angles, with errors usually under 2°. However, it was less accurate for joint range of motion at higher speeds, especially at the hip and ankle. Overall, OpenCap is a convenient tool for gait analysis, but be cautious when looking at ROM at faster speeds.

**What are the main findings?**

- OpenCap showed excellent agreement with MoCap for spatiotemporal gait parameters and continuous joint kinematics across different walking speeds.
- Discrete joint range of motion (especially at the hip and ankle) exhibited lower and speed-dependent reliability, although systematic biases remained small and clinically acceptable.

**What is the implication of the main finding?**

- OpenCap can be reliably applied for gait assessment and monitoring of spatiotemporal and continuous kinematic variables across various walking speeds.
- Clinicians should interpret range of motion outcomes with caution at higher speeds, as their accuracy decreases compared with marker-based measurements.

**Abstract:**

Quantitative gait analysis is essential for understanding motor function and guiding clinical decisions. While marker-based motion capture (MoCap) systems are accurate, they are costly and require specialized facilities. OpenCap, a markerless alternative, offers a more accessible approach; however, its reliability across different walking speeds remains uncertain. This study assessed the agreement between OpenCap and MoCap in measuring spatiotemporal parameters, joint kinematics, and center of mass (CoM) displacement during level walking at three speeds: slow, self-selected, and fast. Fifteen healthy adults performed multiple trials simultaneously, recorded by both systems. Agreement was analyzed using intraclass correlation coefficients (ICC), minimal detectable change (MDC), Bland–Altman analyses, root mean square error (RMSE), Statistical Parametric Mapping (SPM), and repeated-measures ANOVA. Results indicated excellent agreement for spatiotemporal variables (ICC ≥ 0.95) and high consistency for joint waveforms (RMSE < 2°) and CoM displacement (RMSE < 6 mm) across all speeds. However, the joint range of motion (ROM) showed lower reliability, especially at the hip and ankle, at higher speeds. ANOVA revealed no significant System × Speed interactions for most variables, though a significant effect of speed was noted, with OpenCap underestimating walking speed more at fast speeds. Overall, OpenCap is a valuable tool for gait assessment, very accurate for spatiotemporal data and CoM displacement. Still, caution should be taken when interpreting joint kinematics and speed at different walking speeds.

## 1. Introduction

Overground walking in daily life can yield numerous biomechanical insights pertinent to neurological functions and orthopedic outcomes [1]. In this view, quantitative gait analysis plays a central role in clinical decision-making, particularly for initial evaluation and rehabilitation programs that require long-term treatment [2]. Specifically, assessing spatiotemporal parameters and 3D joint kinematics of the lower limbs can provide objective indicators of motor function, disease severity, and treatment response [3,4].

The current gold standard for measuring kinematic variables is 3D motion capture systems (MoCap), which utilize 6–8 high-resolution cameras to provide reliable data on joint angles and velocities. However, the high cost and setup time required, such as the technician’s expertise to properly place the reflective markers, the calibration demands, and the data processing requirements, discourage the most targeted audience from using this procedure. Although new devices have been introduced to the market in recent years to address this issue, utilizing simple mechanisms such as accelerometers [5], they still require costly options for use in daily clinical settings.

These obstacles have spurred the development of markerless, computer vision-based options that estimate human pose directly from video. Recent progress in pose estimation and physics-based modeling shows that markerless systems can achieve the same accuracy as marker-based systems for many locomotor tasks [6]. Among the systems proposed, OpenCap is a markerless pipeline that reconstructs 3D kinematics from synchronized multi-view videos through pose estimation and musculoskeletal modeling [7]. Due to its accessible hardware and streamlined processing, OpenCap enables scalable gait assessment in both clinical and field settings, requiring only smartphones for measurements.

Nevertheless, performance levels assessed by OpenCap technology may vary depending on the population studied [8,9], the setup used for data collection [10], and the specific task demands [11,12,13]. As demonstrated by Martiš et al. [11], who compared walking away and towards the OpenCap system. Although spatiotemporal variables correlated well with the marker-based system during walking in both directions, some joint-angle parameters showed limited agreement when recording was done while walking away from the cameras, resulting in a roughly 12° root mean square error (RMSE). Similarly, Svetek et al. [12] found good agreement in spatiotemporal parameters but observed more errors in hip joint angle assessments during treadmill walking.

Despite these conflicting results, some recommendations regarding gait analysis, such as (i) maintaining an appropriate distance to ensure a good camera angle (30° from the interest area, 3 m away) [7]; (ii) directing gait towards the cameras [11]; and (iii) wearing minimal clothing [10], seem to help researchers reduce overall variability when using specifically OpenCap. Nonetheless, the reliability of measurements at various gait speeds remains a matter of debate. Although markerless systems have shown promising results across different walking and running speeds [14,15,16], their impact in the context of OpenCap has not been explored.

Since walking speed is a key clinical marker for patient evaluation and control during physical therapy [2,17,18,19], interpreting small changes in data can be challenging and lead to errors in speed measurement. To enrich the current findings in the literature related to gait analysis, which focused on fixed spontaneous walking speeds only [7,8,10,11,12,20], the present study aimed to evaluate the agreement between OpenCap and a marker-based system during walking at three different speeds: slow, self-selected (self), and fast. By establishing accuracy across various walking speeds, this work aims to support the practical application of OpenCap for gait assessment, focusing on spatiotemporal variables and lower-limb joint angles, in routine clinical practice.

## 2. Materials and Methods

### 2.1. Participants

Fifteen healthy participants (7 females and 8 males), all without musculoskeletal injuries, with an average age of 32.3 ± 8.3 years (mean ± SD), a height of 1.74 ± 0.1 m, and a weight of 71.1 ± 21.6 kg, were enrolled in this study. Participants were required to be adults who could ambulate independently, with no history of neurological, cardiovascular, or musculoskeletal disorders that could affect their gait, and no lower-limb surgeries or injuries. All demonstrated a regular gait pattern without significant functional deficits. The experimental procedure was carried out following the standards laid down in the Declaration of Helsinki and approved by the “Comitato Etico di Ateneo dell’Università degli Studi di Milano” (council 23/23).

### 2.2. Experimental Setup

The experimental session involved analyzing walking on the ground at three different speeds. Before beginning each trial, they completed a series of walks until they felt confident and comfortable with their self, faster, and slower speeds (respectively), each lasting between 2 and 3 min. The self-speed served as a baseline for the fast and slow speeds. The order of the tests was fixed for all participants. First, the self-selected speed, followed by the fast and slow speeds, respectively. This standardized sequence was selected so that each participant could use their own speed as a reference point for the other two conditions. The fast and slow conditions were therefore defined subjectively by each participant and visually verified by the duration of each trial. For the fast condition, participants were asked to walk slightly faster than their self-paced speed, and for the slow condition, they were asked to walk marginally slower than their self-paced speed. It did not impose a fixed percentage of variation, allowing participants to determine these speeds themselves to ensure a natural gait pattern.

For each speed, participants were instructed to walk as naturally as possible in an 8-m track after hearing a verbal prompt. Verbal feedback on their rhythm was provided at the end of each trial.

All participants wore minimal clothing, including shorts and sports shoes for walking, and a sports bra for females.

### 2.3. Recordings

**Figure 1:**
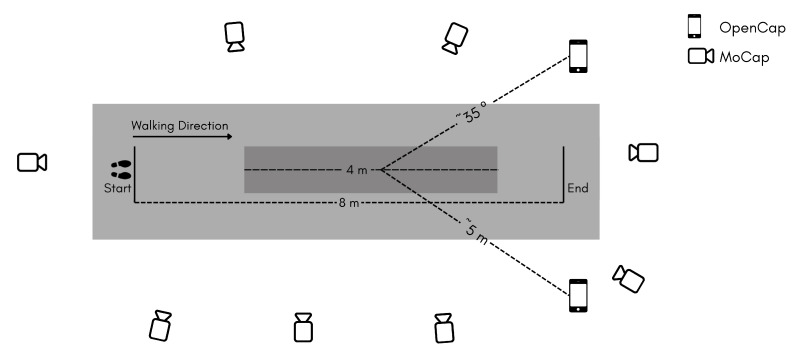
Laboratory setup for both systems, MoCap and OpenCap, during the test. The darker gray area represents the 4-m walkway in OpenCap’s acquisition volume.

Data were collected simultaneously using a MoCap and an OpenCap system (Figure 1). Participants walked along an 8-m walkway, but only the central 4 m were analyzed. This region was chosen because it corresponded to the adequate capture volume of the OpenCap system (4 × 4 m), spanning ± 2 m from the calibration point, and ensuring the most natural walking possible, which matched the optimal field of view of the two OpenCap cameras. The two systems were synchronized in time using a light signal emitted by a handheld flashlight, given before each walking trial began. This synchronization was used solely to pair corresponding strides across systems. Considering the frame rates of each auxiliary video (25 Hz for MoCap and 120 Hz for OpenCap), the maximum possible temporal mismatch after alignment was approximately ±0.04 s.

#### 2.3.1. MoCap

**Figure 2:**
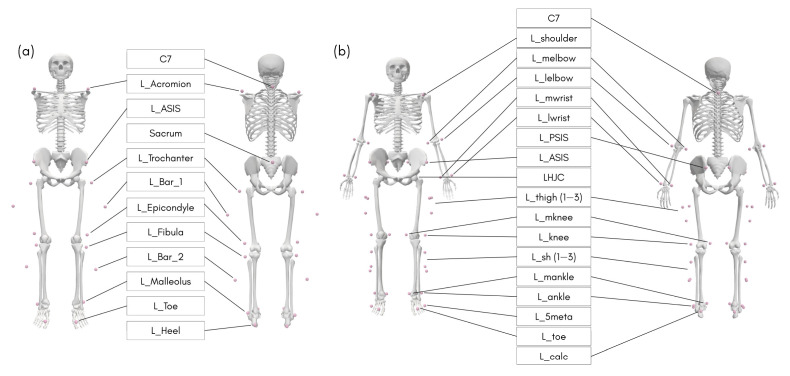
Representative models of MoCap (**a**) and OpenCap (**b**) systems in anterior and posterior views. The markers are listed for MoCap as the seventh cervical vertebra (C7), acromion (Acromion), anterior superior iliac spine (ASIS), sacrum (Sacrum), greater trochanter (Trochanter), upper lateral marker on the rigid bar attached to the thigh (Bar_1), lateral femoral epicondyle (Epicondyle), mid-lateral shank (Fibula), lower lateral marker on the rigid bar attached to the shank (Bar_2), lateral malleolus (Malleolus), head of the second metatarsal (Toe), and posterior calcaneus (Heel). The markers are listed for OpenCap as the seventh cervical vertebra (C7), acromion (shoulder), medial humeral epicondyle (melbow), lateral humeral epicondyle (lelbow), medial wrist styloid process (mwrist), lateral wrist styloid process (lwrist), posterior superior iliac spine (PSIS), anterior superior iliac spine (ASIS), hip joint center (HJC), three markers distributed along the lateral thigh (thigh 1–3), medial femoral epicondyle (mknee), lateral femoral epicondyle (knee), three markers distributed along the lateral shank (sh 1–3), medial malleolus (mankle), lateral malleolus (ankle), head of the fifth metatarsal (5meta), head of the second metatarsal (toe), and posterior calcaneus (calc).

An optoelectronic MoCap system with eight cameras (SMART DX 100, BTS Bioengineering SPA, Milan, Italy) was used, set to record data at a rate of 100 Hz. Twenty-two reflective markers were placed on anatomical landmarks following the model described by Davis et al. [21] (Figure 2a). The system was calibrated according to the manufacturer’s recommendations, and a static calibration trial was performed with the participant in a standing position. Raw kinematic trajectories were reconstructed using the system’s proprietary software (SMART-Clinic, BTSBioengineering SPA, Milan, Italy) and exported in *.trc* format for further processing and analysis.

#### 2.3.2. OpenCap

Simultaneously, data for the OpenCap system were collected using two iOS smartphones (iPhone 13 and iPhone 13 Pro, Apple Valley, CA, USA), each mounted on a tripod. The cameras were positioned approximately 35° relative to the central axis of the walkway, with the lenses 1.2 m above the ground and perpendicular to the floor, about 5 m from the calibrator. A system calibration was performed following the developer’s recommendations, and a static calibration trial was conducted with the participant in a standing position. Videos were recorded at 120 Hz and processed in the cloud using the HrNet pose estimation algorithm, with default settings recommended by OpenCap. The musculoskeletal model used was the default Full Body model, based on the models proposed by Lai et al. [22] and Rajagopal et al. [23], with modifications to the hip abductor muscle trajectories as described by Uhlrich et al. [7] (Figure 2b). The OpenCap version used was from July 2025, and all procedures adhered to the developer’s recommendations and best-practice guidelines available at www.opencap.ai (accessed on 01 July 2025).

### 2.4. Raw Data Processing

The reconstructed marker trajectories from the MoCap system were used to perform inverse kinematics in OpenSim. For model scaling, the marker set from the adopted model was applied to the musculoskeletal model [23]. Scaling was carried out according to each participant’s anthropometric proportions, and marker positions were adjusted based on data collected during a static trial (i.e., calibration). Before applying inverse kinematics, the 3D marker trajectories were low-pass filtered using an 8 Hz Butterworth filter, as implemented in OpenSim, to reduce measurement noise and prevent spurious peaks in the subsequent joint angle calculations. After completing the inverse kinematics, the motion data were exported and analyzed in OpenSim using the Analysis tool with the BodyKinematics module to determine the position of the center of mass (CoM).

OpenCap automatically performs inverse kinematics and, after download, provides three-dimensional marker position files (.*trc*), joint angle data (.*mot*), and the scaled model customized to the individual’s anthropometrics. To determine the CoM position, these data were processed in OpenSim using the *BodyKinematics* tool. OpenSim version 4.5 for macOS was used.

The synchronization between systems was done manually using videos recorded by both MoCap and OpenCap. The videos were loaded into a media player, and the frame showing the flashlight flash was identified and used as the reference point to synchronize the events recorded by both systems in time.

With the *.trc*, *.mot*, and CoM position files saved, along with the identified synchronization times, the data were imported into a custom routine developed in Python 3.12. All signals were interpolated to 100 Hz and low-pass filtered using a second-order Butterworth filter with a cutoff frequency of 6 Hz. The native sampling frequencies were 100 Hz for MoCap and 120 Hz for OpenCap; the latter was resampled to 100 Hz using linear interpolation to enable direct comparison. We chose linear interpolation because it is a reliable and simple method commonly used in gait analysis when the temporal resolution is high, and the risk of aliasing is minimal. The 6 Hz cutoff was chosen following the consolidated practice in gait biomechanics and has been consistently adopted, providing an effective balance between noise reduction and signal preservation [24].

Marker positions were used to identify touchdown (TD) and take-off (TO) events following the protocol described by Zeni et al. [25]. Specifically, TD was defined as the frame in which the heel/calc (MoCap/OpenCap) marker reached its most forward position relative to the pelvis, while TO was defined as the frame in which the toe marker reached its most backward position relative to the pelvis. These events were used to segment strides and compute spatiotemporal parameters, along with joint angular data. Only events with corresponding detections in both systems were analyzed, and those without simultaneous matches were excluded.

There were no marker losses during MoCap recordings, and OpenCap successfully reconstructed all key joints without issues. Additionally, all automatically detected TD and TO events were visually inspected to confirm the correct sequence of strides.

All processed data were saved in an Excel file (.xlsx), organized into multiple sheets containing raw data, processed outputs, stride-averaged summaries by participant, and interpolated continuous curves for subsequent statistical analysis.

### 2.5. Experimental Variables

#### 2.5.1. Spatiotemporal

The protocol by Zeni et al. [25] for identifying gait events, which relies on the forward and backward movement of heel and toe markers, served as the basis for our spatiotemporal analysis results.

A stride is the period between two successive TD events of the same foot. Stride Time measures the interval between these events, and Stride Length is derived from the heel marker’s anterior–posterior displacement during that period. Double Support Time is the interval from the TD of one foot to the TO of the opposite foot, indicating when both feet contact the ground simultaneously. Cadence is calculated by dividing the total number of steps by the analysis duration, expressed in steps per minute. Walking speed was determined by dividing the total front–back displacement of the virtual mid-hip marker by the total recording time, which is the elapsed period between the first and last detected foot-contact events within each trial. The choice of mid-hip represents a standardized point available in both MoCap and OpenCap systems.

#### 2.5.2. Joint Angles

Joint angle variables were derived from the joint kinematics files generated by OpenSim (*.mot*) for both the MoCap and OpenCap systems. Joint angle waveforms were segmented based on the previously defined stride events, considering complete cycles between two consecutive touchdowns of the same limb. Each cycle was interpolated to 101 points using linear interpolation, representing 0–100% of the gait cycle in normalized form. From these continuous curves, three discrete variables were calculated for each joint: the minimum value (Min), the maximum value (Max), and the range of motion (ROM), defined as the absolute difference between the maximum and minimum values observed within the cycle, averaged across all valid strides of each participant within each condition.

Analyses were performed separately for each joint (hip, knee, and ankle), with all the angular variables assessed in the sagittal plane. Since participants were healthy and did not present any clinically perceptible gait asymmetry, values from the left and right limbs were averaged to reduce redundancy and obtain a single representative measure per variable.

### 2.6. Statistics

Data normality was verified using the Shapiro–Wilk test, with no violations detected (*p* > 0.05). Accordingly, descriptive statistics were presented as means and standard deviations (SD), and parametric methods were used for comparisons [26]. Agreement between systems for the discrete variables (stride length (m), stride time (s), double support (s), cadence (Hz), walking speed (km.h^−1^), hip ROM (°), knee ROM (°), and ankle ROM (°)) was evaluated using multiple complementary approaches.

We generated Bland–Altman plots with 95% limits of agreement (LoA) to assess systematic bias and the range of agreement. Reliability was measured using the intraclass correlation coefficient (ICC, model 2,1), while Pearson’s correlation coefficient was used to analyze linear association. We included both metrics because they offer complementary insights: the ICC evaluates absolute agreement between systems, whereas Pearson’s r measures the strength of the linear relationship regardless of constant differences. Although the ICC is generally more useful for agreement, Pearson’s r was also included to facilitate comparison with previous validation studies, which often report correlations. Furthermore, the minimal detectable change (MDC) at the 95% confidence level was calculated to indicate the smallest difference that can be detected beyond measurement error. This MDC was calculated from the standard error of measurement (SEM), which we estimated from the standard deviation of the between-system differences.

For continuous joint angles data (i.e., hip, knee, and ankle) and CoM displacement, agreement was additionally examined by calculating the average magnitude of the differences between predicted and actual values using the root mean square error (RMSE) and performing Statistical Parametric Mapping (SPM) to identify regions of the gait cycle with statistically significant differences. SPM analyses were performed separately for each sagittal joint angle (hip, knee, and ankle) using paired *t*-tests (α = 0.05), considering all strides from all participants. Significant intervals were highlighted in the plots, and for each of these regions, we also reported the local RMSE. The overall RMSE values were calculated by combining the data from all strides and participants. RMSE values below 5° for angular variables were considered indicative of good agreement, following previously established clinical thresholds [27,28].

To determine whether differences across walking speeds were consistent within each system and to evaluate a System × Speed interaction, we conducted a 2 × 3 repeated-measures ANOVA with within-subject factors system (MoCap, OpenCap) and speed (slow, self, fast). Sphericity was tested with Mauchly’s test; if violated, Greenhouse–Geisser (G-G) corrections were applied. Significant effects were followed by Bonferroni-adjusted simple-effects comparisons: (i) speed differences within each system and (ii) system differences at each speed. We selected the Bonferroni correction due to its simplicity and strong control of type I error. In addition to F and *p* values, we also reported partial eta squared (ηp^2^) as a measure of effect size for main effects and interactions.

We quantified the between-system error for each discrete variable as Δ = OpenCap − MoCap computed per trial. The Δ was regressed on MoCap walking speed (km.h^−1^) using ordinary least squares with speed mean-centered for interpretability. We confirmed the adequacy of the linear model by testing a quadratic term, which was not significant across all variables (*p* ≥ 0.12, Δ AIC < 2), and by conducting the Ramsey RESET test, which showed no evidence of model misspecification (*p* ≥ 0.19). These results support the use of a linear model, for which regressions were conducted at the trial level. We report the slope (two-tailed α = 0.05), Pearson’s r, and R^2^; a positive slope indicates an increasing overestimation of OpenCap with higher speed (proportional bias).

All statistical analyses were carried out using JASP (version 0.95) and Python (version 3.12). In Python, the following packages: NumPy (v1.26), pandas (v2.2), SciPy (v1.13), statsmodels (v0.14), pingouin (v0.5), and spm1d (v0.4) were used for descriptive statistics, repeated-measures ANOVA, post hoc tests, effect size estimation, and Statistical Parametric Mapping (SPM) analyses.

## 3. Results

In the overall dataset, each participant completed an average of 3.8 ± 1.1 walking trials (mean and ± SD), totaling 169 trials. The average number of detected strides was 6.7 ± 3.4 (mean and ± SD), with 297 strides analyzed in total. The overall average walking speed was 4.7 ± 1.1 km.h^−1^ (mean and ± SD). When divided by speed, the slow, self, and fast groups had, respectively, 3.6 ± 1.1, 3.9 ± 1.4, and 4.0 ± 1.0 trials per participant (mean and ± SD). The mean number of strides per participant was 8.4 ± 3.4, 4.7 ± 2.4, and 7.0 ± 3.4 (mean and ± SD) for the slow, self, and fast conditions. In total, 60 trials and 126 strides were recorded for the slow condition, 58 trials and 105 strides for self, and 51 trials and 66 strides for the fast condition. The mean speeds for each category were 3.6 ± 0.5 km.h^−1^, 4.6 ± 0.4 km.h^−1^, and 6.1 ± 0.5 km.h^−1^, respectively.

The agreement results regarding the spatiotemporal and joint angle parameters were divided and presented as discrete and continuous angular variables. For each, the agreements were shown stratified by speed. Additionally, the last section examines the relationship between MoCap walking speed and the between-system error for each spatiotemporal and kinematic variable across speeds.

### 3.1. Agreement for Discrete Variables

#### 3.1.1. Speed Condition Agreement

**Figure 3:**
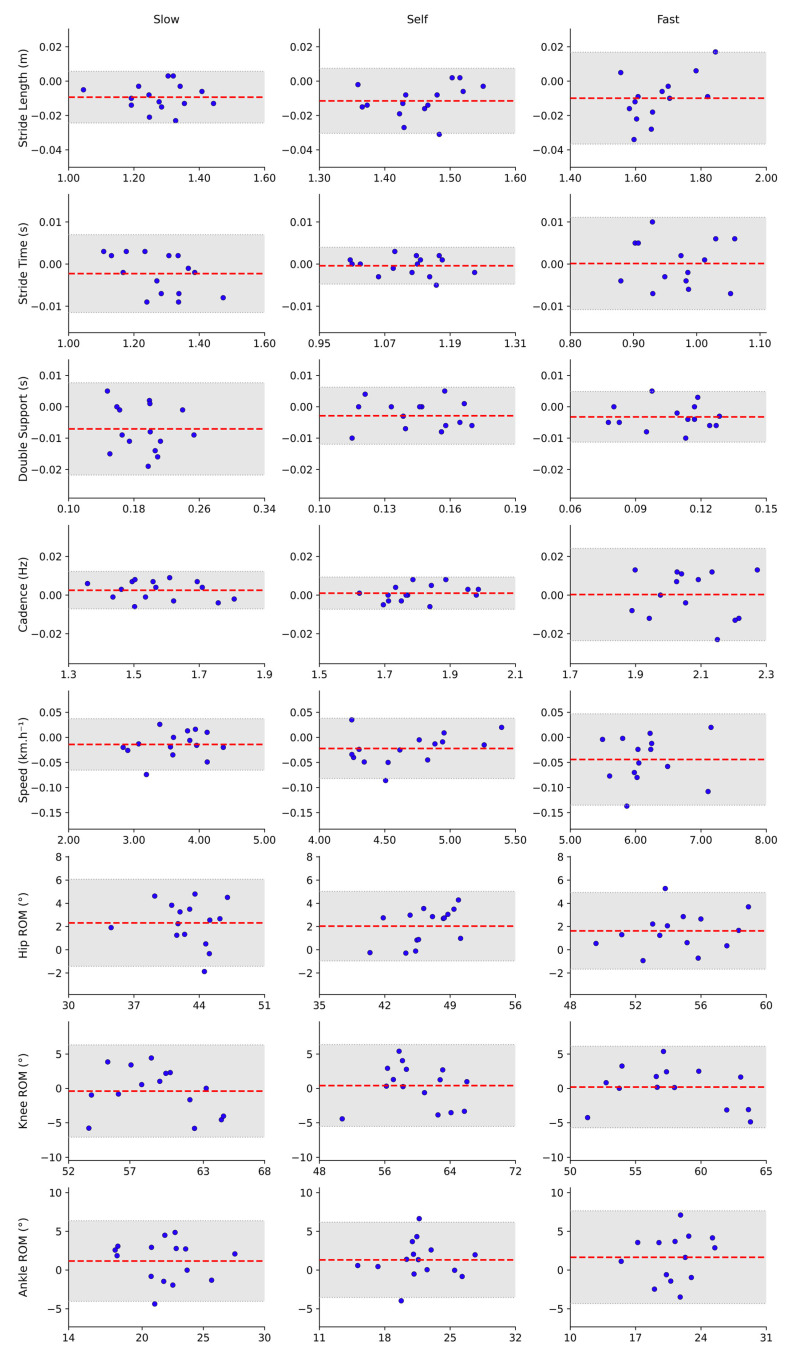
Bland–Altman plots illustrating the agreement between MoCap and OpenCap across various speeds. Each subplot displays the difference between the systems (*y*-axis) versus the mean of both systems (*x*-axis) for a specific variable, separated by speed: Slow (**left**), Self (**Center**), and Fast (**Right**). The red dashed line indicates the mean bias between methods. The gray shaded area shows the 95% limits of agreement (LoA), while the dotted lines mark the upper and lower LoA boundaries.

The stratified analysis by speed showed that spatiotemporal variables had excellent agreement between systems across all conditions (ICC ≥ 0.95; r ≥ 0.967) (Table 1), with mean biases close to zero (Figure 3) and low MDC values (0.004–0.09), indicating high precision and stability of measurements regardless of speed. In contrast, the reliability of the ROM of angular variables was less consistent, showing low to moderate ICCs (0.482–0.776), mild to strong correlations between the systems (0.54–0.89), and higher MDC values (2.99–6.71), indicating greater overall variability and lower reproducibility for all joints, especially more prominent at the ankle during higher walking speeds.

#### 3.1.2. Speed vs. System Interaction and Effects

The repeated measures ANOVA did not show any interaction between the system (MoCap, OpenCap) and speed (slow, self, fast) for all variables except for walking speed (km.h^−1^) (G-G corrected *p* = 0.00030; ηp^2^ = 0.50).

Across systems, speed displayed strong main effects for all spatiotemporal variables—cadence, stride time, stride length, double support, walking speed—and for hip ROM (all *p* < 0.001; ηp^2^ ≈ 0.88–0.95 for spatiotemporal and ηp^2^ = 0.92 for hip ROM). Only knee ROM showed a smaller but significant effect of speed (G-G corrected *p* = 0.027; ηp^2^ = 0.264), whereas ankle ROM did not change with speed for either system (*p* = 0.39; ηp^2^ = 0.07).

Amongst speeds, the system’s effects had strong significance in double support (ηp^2^ = 0.48), stride length (ηp^2^ = 0.57), walking speed (ηp^2^ = 0.40), and Hip ROM (ηp^2^ = 0.70), with all *p* < 0.05. The other variables, such as cadence, stride time, and knee ROM, were not significant (*p* > 0.05), with minor effects (ηp^2^ < 0.01). The only exception was the ankle ROM, which was not significant due to the system effect, but showed a higher effect size (ηp^2^ = 0.22).

### 3.2. Agreement for Continuous Joint Angle Variables

#### Speed Condition Agreement

**Figure 4:**
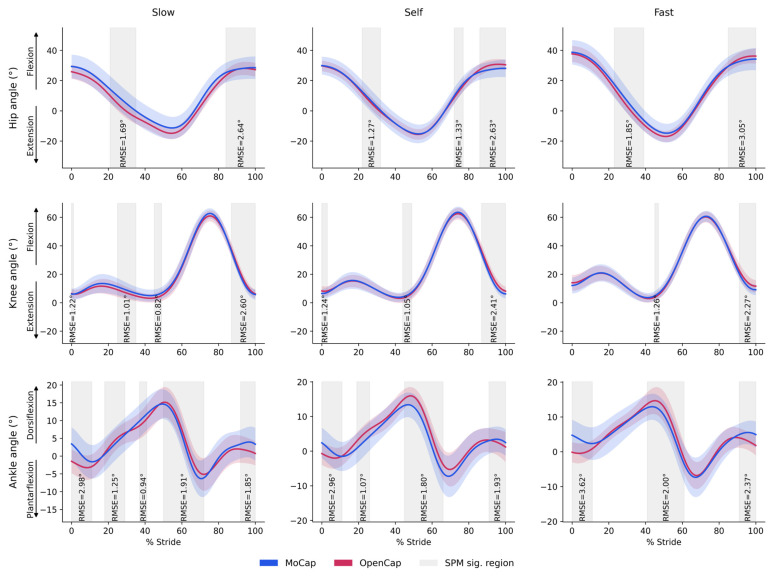
Joint angles across the gait cycle at three speeds for MoCap and OpenCap systems. Mean ± SD of hip, knee, and ankle joint angles over the normalized gait cycle (% stride) for MoCap (blue) and OpenCap (red) at slow, self, and fast speeds. Gray-shaded regions indicate phases with statistically significant differences between systems (SPM sig. region) with corresponding RMSE values shown for each highlighted region.

The results showed global RMSE values below 2° for all assessed joint angles, except for specific moments highlighted in the shaded regions. The ankle presented the highest mean RMSEs (1.77° in Fast, 1.52° in SS, and 1.59° in Slow), possibly reflecting greater sensitivity of this joint to slight differences in event detection and distal segment positioning. The hip and knee exhibited smaller discrepancies between systems, with RMSEs ranging from ~1.00° to 1.52° (Figure 4).

**Figure 5:**
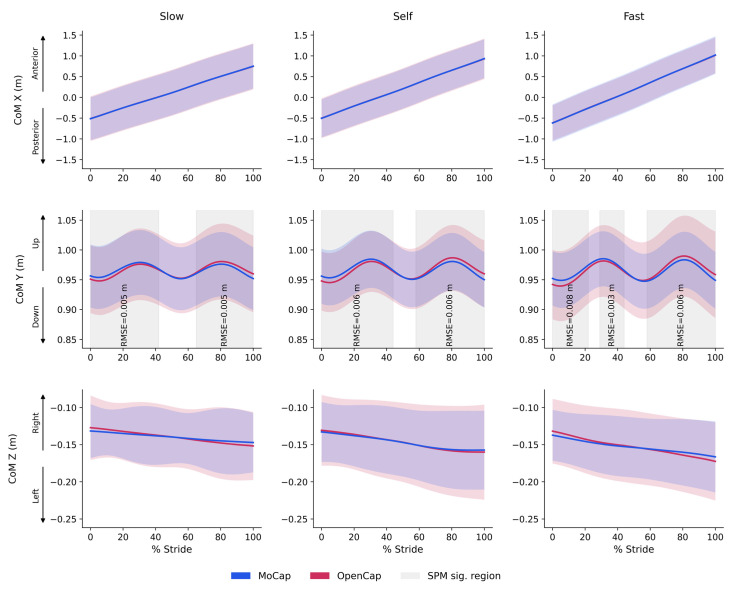
CoM trajectories across the gait cycle at three walking speeds for MoCap and OpenCap systems. Mean ± SD of CoM displacement in the mediolateral (X), vertical (Y), and anteroposterior (Z) directions over the normalized gait cycle (% stride) for MoCap (blue) and OpenCap (red) at slow, self, and fast speeds. Gray-shaded regions indicate phases with statistically significant differences between systems (SPM sig. region) with corresponding RMSE values shown for each highlighted region.

The calculation of the CoM displacement demonstrated high agreement between systems, with root mean square errors (RMSE) of less than 6 mm in all directions and speeds. The vertical axis (CoM Y) presented the highest absolute values, although the maximum observed difference was only 0.006 m. The anteroposterior (CoM X) and mediolateral (CoM Z) displacements showed slightly smaller errors (0.002–0.003 m) (Figure 5).

### 3.3. Relationship and Differences Between Walking Speeds and Between-System Error

**Figure 6:**
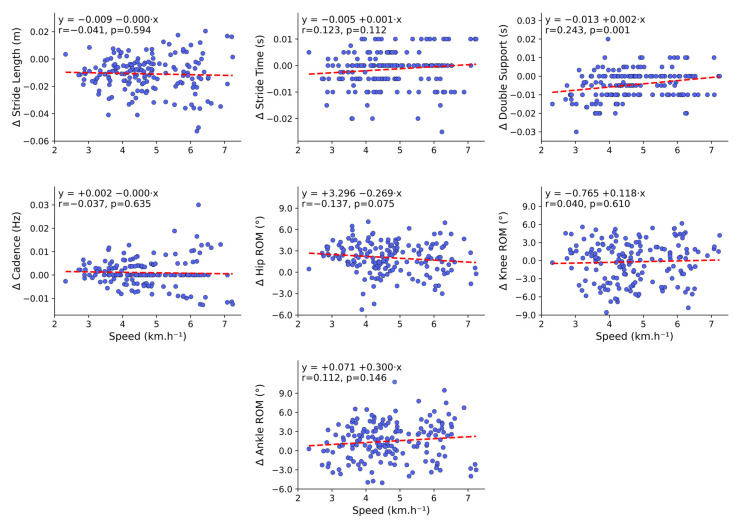
Relationship between MoCap walking speed (km.h^−1^) and between-system error in spatiotemporal and kinematic variables. Scatterplots of the between-system error (*y*-axis) for each variable, plotted against MoCap walking speed (km.h^−1^; *x*-axis). The red dashed line is the trend line output of the least-squares fit (y = b + a.x); subplot headers report Pearson’s r and *p*.

The association between MoCap walking speed (km.h^−1^) and the between-system error showed very low coefficients of determination (R^2^ ≤ 0.059) for all variables, indicating no significant linear relationship. Most parameters had *p*-values above the significance threshold (*p* > 0.05), except for double support (s), which showed a statistically significant association (*p* = 0.001) but with a weak effect (R^2^ = 0.059). Overall, the between-system error remained consistent across walking speeds, with no evidence of speed-dependent (proportional) bias and only minor, non-significant trends (Figure 6).

## 4. Discussion

The primary analysis of this study demonstrates how biomechanical variables captured by a markerless system (OpenCap) during walking at different speeds align with those from a gold-standard marker-based system (MoCap). To our knowledge, this is the first research to examine these variables across various walking speeds.

### 4.1. Agreement for Discrete Variables: Spatiotemporal Parameters

Building on previous research, the OpenCap system closely aligns with the MoCap system in terms of spatiotemporal gait variables during walking, particularly at different speeds. Mean biases are close to zero, with high intraclass correlation coefficients and low minimal detectable changes, which supports the reliability of these parameters in the markerless system approach. However, analysis of discrete joint ranges of motion exhibited more variability, especially in hip and ankle movements, where reproducibility ranged from moderate to low. In contrast, continuous kinematic waveforms and CoM displacement demonstrated high consistency between systems over the different speeds proposed, with smaller mean errors.

When analyzing the spatiotemporal data, recent validation studies of OpenCap during overground walking have reported findings consistent with our results, particularly for spatiotemporal variables [7,11,29]. In our study, we observed an almost perfect agreement between OpenCap and MoCap for stride length and stride time, cadence, double support time, and walking speed, with mean biases close to zero, high intraclass correlation coefficients (ICCs), and consistently low minimal detectable changes (MDCs) (for details, see Table 1). This high reliability can be attributed to the robustness of pose estimation algorithms in identifying distal landmarks (e.g., the heel and toes), which serve as direct inputs for calculating events such as touchdown and takeoff [25]. Unlike angular kinematics, which depend on complex estimation of 3D joint centers and segment orientations, spatiotemporal parameters are less vulnerable to errors from camera angles or partial occlusions [7]. These results are consistent with the literature; for example, Martiš et al. [11] found very high correlations (r ≈ 0.94–1.0) at an average walking speed, while Horsak et al. [10] and Uhlrich et al. [7] reported excellent validity during ‘self-selected’ natural walking similar to our self-selected speed (~4.5 km.h^−1^). Our findings confirm high reliability, not only at a self-selected, but also at slower and faster speeds. Additionally, our results align with those of Uhlrich et al. [7], who highlighted the strong accuracy of OpenCap for these parameters, not only during walking but across various protocols, including squatting, rising from a chair, and drop jumps, further supporting the system’s reliability for these types of variables.

### 4.2. Agreement for Discrete Variables: Range of Motion Parameters

In contrast, for discrete ROM analysis, both our results and the existing literature show greater limitations. In this study, all the joints presented low to moderate agreement (ICCs ~ 0.48–0.78), with relatively high minimal detectable changes (MDCs) (e.g., 3–7° for all speeds). Precisely, only the knee demonstrated a moderate to strong edge agreement over all walking speeds (ICC ~ 0.65–0.78, MDC ~ 5.9–6.7°). Such a difficulty in reporting higher reliability for joint ROMs was noted by Turner et al. [28], who found good validity for hip and knee motion in the sagittal plane but lower reliability for the ankle during uni- and bilateral movements, as well as jumping. Comparable findings were reported by Horsak et al. [10], who, in a repeatability study of OpenCap, identified the hip and ankle as the joints with the highest variability and noted high MDC values for both, further emphasizing them as challenging joints for this system. Other tasks, such as landing, showed that the hip and ankle remain the joints most prone to errors [30]. Even though continuous joint angle waveforms show high similarity between systems (RMSE < 2° in our case), localized differences in specific phases of the gait cycle, as revealed by our SPM analysis, can shift minimum or maximum values. Since the calculation of discrete ROM relies solely on these two extreme data points, it is inherently more vulnerable to localized errors. In contrast, metrics like RMSE are based on averaging, which spreads the error across the entire gait cycle and can mask these brief, peak-related discrepancies. This difference in calculation methods may explain why discrete ROM measures exhibit lower reproducibility, even when the underlying continuous waveforms appear highly consistent (see Figure 4).

Also, despite constant algorithm system updates, it is worth noting that the pose estimation model continues to struggle with detecting markers from discrete angle joints, which are limited by sparse and noisy keypoints [31]. Aiming to improve marker detection for enhancing accuracy and generalizability for walking and other motor tasks, Falisse et al. [31] recently integrated a Long Short-Term Memory (LSTM) model into OpenCap, which predicts the 3D position of 43 denser anatomical markers based on the 3D position of 20 sparser keypoints. In their study, the new marker enhancer decreased the overall RMSE (error metric) of kinematic angle joints compared to a marker-based system, from 5.3° (older OpenCap system) to 4.1° (LSTM model). In our case, despite the already integrated LSTM model in OpenCap, the higher MDC reported in the ROM from the lower limb joints at all measured speeds still shows the difficulty for their developers to enhance the system in this situation.

Furthermore, the limited volume calibration used for OpenCap could worsen the higher metric errors observed in the discrete ROM variables, as only a small number of trials were conducted. Although designed to mimic natural walking over the ground, this approach may result in less data being analyzed compared to treadmill trials, even though, in this case, the data quality depends on the position of the safety bars [12]. Additionally, from this methodological standpoint, our study meets the highest standard possible by using OpenCap during walking. The use of two cameras at front-side strategic angles can cover the largest possible area without significant errors [7]. Currently, the best results from this markerless system are achieved either in this way or when the model is updated.

Unlike spatiotemporal variables, for which normative values can be established to detect minimal clinical differences (see Section 4.5), variables such as discrete joint ROMs have not yet been standardized due to the unpredictable behavior of pathological gait [32,33]. For example, when comparing the MDC for hip flexion-extension ROM obtained with OpenCap and MoCap systems (2.99°) to the Minimal Clinically Important Difference (MCID) recently reported for chronic stroke patients (5.81° for the affected and 2.86° for the unaffected limb; Guzik et al. [34], our results show that the system’s measurement variability stays below the level of clinically meaningful change. This indicates that the markerless system can detect improvements in hip sagittal motion that are relevant for rehabilitation. The MDC, which accounts for approximately 51% of the MCID for the affected limb, further confirms the system’s sensitivity to key changes in hip movements. Along with the knee results, where the MDC (5.96°) was lower than the MCID values reported by Guzik et al. [35] (6.8–8.5°), these findings verify that OpenCap’s measurement accuracy for major lower-limb joints is within clinically acceptable ranges. Therefore, even small rehabilitation-related changes in sagittal plane kinematics can be reliably identified by the markerless system for stroke patients.

### 4.3. Agreement for Continuous Variables: Center of Mass Displacement

In the analyses of continuous joint angle waveforms, the RMSE values were consistently low across all walking speeds (<2° for the hip, knee, and ankle), indicating high similarity between joint profiles and remaining below the acceptable thresholds for gait variables [27]. This result reinforces previous findings with OpenCap, which also reported reduced discrepancies between marker-based and markerless systems, particularly for sagittal plane joints [7,10]. Moreover, our continuous joint angle values across all walking speeds were considerably low, with global RMSEs ranging from ~1.00° to 1.77°. This indicates a higher accuracy than that reported in prior gait studies with OpenCap, where RMSE values generally ranged between ~4° and 7° [11,29]. Similarly, the analysis of CoM displacement showed high agreement between systems, with an RMSE of less than 6 mm in all directions and speeds. The vertical axis (CoM Y) presented the largest absolute values, although the maximum difference was only 0.006 m. The anteroposterior (CoM X) and mediolateral (CoM Z) displacements exhibited even more minor errors (0.002–0.003 m). These findings confirm that, in addition to joint angles, OpenCap also provides reliable estimates of CoM trajectories, even at different overground walking speeds. This result, consistent with previous findings, has been reported in validation studies of markerless systems compared to marker-based methods, demonstrating high accuracy for CoM trajectories during self-selected walking [36].

Furthermore, the initial OpenCap study also emphasized the accuracy of this method in capturing three-dimensional CoM displacement, providing reliable measurements [7]. This robustness, observed in our study even at different speeds, can be attributed to the calculation method: OpenCap provides the input kinematic data (i.e., the 3D position of each body segment), which is then utilized by the OpenSim software for biomechanical analysis. Within OpenSim, the CoM is a model-based calculation derived from a full-body musculoskeletal model scaled to each participant’s anthropometry. This method computes the total-body CoM as a mass-weighted average of all individual segments. This integrative approach makes the CoM calculation less sensitive to minor errors [37].

### 4.4. Agreement for Continuous Variables: Range of Motion Parameters

The use of SPM was essential to interpret the results from the continuous joint angle waveforms, as we identified localized differences at specific points of the gait cycle (e.g., near toe-off), but without a relevant impact on the overall mean error (~1.00° to 1.77°) (see Figure 4). Previous studies with OpenCap have also reported discrepancies at critical events, such as initial contact and take-off [11,30]. Taken together, these findings emphasize that even when overall RMSE values are low, local discrepancies can still account for errors in specific measures, such as ROM.

### 4.5. Relationship and Differences Between Walking Speeds and Between-System Error

Additionally, when examining the relationship between walking speeds and the between-system error, represented by the error deltas (Δ), these relationships were all low (R^2^ ≤ 0.059) for all variables. In other words, the between-system error for spatiotemporal parameters (e.g., stride length, cadence) and all discrete joint ROMs (hip, knee, and ankle) remained consistent across the tested walking paces, with no trend toward greater errors at higher speeds. These findings, suggesting the absence of proportional bias for most variables, align with a growing body of evidence demonstrating high agreement for spatiotemporal parameters in markerless systems, both at self-selected speeds [29] and across different walking speeds [16,38,39]. The only variable with a distinct behavior was double support time, which exhibited a statistically significant but very weak correlation with speed (*p* = 0.001, R^2^ = 0.059). Evidence in the literature corroborates this greater sensitivity of double support, as it represents a brief interval in which both feet are in contact with the ground, making it more prone to discrepancies between analytical methods, with accuracy varying according to walking speed (~78.7% to 84.5%) [40].

The repeated-measures ANOVA (system × speed) reinforced this pattern. For both systems, the speed exerted robust main effects on all spatiotemporal variables—cadence, stride time, stride length, double support, and walking speed—as well as on hip ROM (all *p* < 0.001; and large ηp^2^ between 0.88 and 0.95 for spatiotemporal variables and 0.92 for the hip). This behavior is expected and widely described in the gait literature [24,41,42]. Knee ROM also varied significantly with speed, albeit with a more negligible effect (*p* = 0.027), but still supported by larger effect sizes (ηp^2^ = 0.264). Here, the interaction between speed and system was not significant (*p* = 0.12), but the strong effect size (ηp^2^ = 0.16) raises concerns and practical relevance regarding the speed magnitudes that influence differences between OpenCap and Mocap.

In contrast, ankle ROM remained unchanged with increased speed (*p* = 0.39). This lack of significant impact on the ankle should be interpreted with caution, given that the effect size was relatively weak (ηp^2^ = 0.07). Otherwise, given the lower reliability and higher measurement variability we previously identified for discrete ankle ROM (ICCs as low as 0.482), it would be plausible that this measurement noise might have masked a true, albeit smaller, physiological effect of speed on this joint. Even when reporting the impact of speed for both systems (the average between OpenCap and Mocap), this result still highlights the difficulty of the OpenCap system in detecting differences in walking speeds at distal joints, particularly at the ankle ROM.

It is also worth noting that no significant interactions were found between systems (MoCap, OpenCap) and speed (slow, self, fast) for any variable, except for walking speed (km.h^−1^). Post hoc analysis showed that OpenCap estimated slightly lower values than MoCap at all speeds (differences that increased with speed, from −0.014 and −0.046 km.h^−1^), but of minimal magnitude, a pattern similar to that reported by other authors [11,40]. Methodologically, while MoCap calculates speed using physical markers on the feet and pelvis, OpenCap depends on musculoskeletal models and computer vision-based reconstruction. This methodological difference could explain the results, which show a slight but consistent underestimation of the forward displacement of the mid-hip, likely contributing to the significant bias and larger effect size (ηp^2^ = 0.50) in the lower velocity measurement when the actual speed increases. This indicates that although there is a minimal systematic bias, both systems equivalently capture the relative changes in walking speed.

In addition, from a practical point of view, the Minimal Clinically Important Differences (MCID) may reinforce such a behavior [32,33]. Indeed, Bohannon et al. [33] reported a range of 0.36–0.61 km.h^−1^ for detecting minimal significant differences at comfortable speeds in the clinical environment. Replicating our results and considering the self-selected speed as the comfortable one, the underestimation by OpenCap of the Self speed (i.e., the 5 km.h^−1^ recorded would actually be about 5.03 km.h^−1^, a roughly 0.03 km.h^−1^ error) would account for 8.3% of the minimal detectable difference reported by the authors (0.36 km.h^−1^). This means that even subtle speed differences, which were notably reported between OpenCap and Mocap systems in the present study, would not have a significant clinical impact. The same interpretation might be extended to slow and fast speeds, which had errors ranging from 4.7% to 13.9%, respectively, related to those presented by Bohannon et al. [33] for indicating actual clinical differences.

Despite the overall strong agreement, some possible sources of error need attention. Double support time, for instance, although there was no interaction between speed and system (*p* = 0.09), a strong effect size (ηp^2^ = 0.19) was reported, which could indicate differences between the systems as speed increases. In fact, this result would not be surprising, as it relies on accurately detecting contact events; therefore, slight shifts in the virtual markers could introduce bias [11,29]. In addition, methodological differences between systems may influence walking speed, as factors such as pelvis estimation and camera angle directly affect this measurement [11,29]. Therefore, the interaction observed for walking speed appears to reflect systematic biases related to modeling and capture, rather than a limitation in the system’s ability to detect speed changes.

### 4.6. Limitations

A key limitation is the limited capture volume of the OpenCap system, which restricts data collection to a walking distance of about 4 m. As a result, only a small number of strides could be recorded per trial. It would not represent a flaw in our experimental design but an inherent limitation of the system itself. While repeating trials helps reduce this issue, it may still affect the representativeness of gait dynamics over more prolonged walking bouts.

Furthermore, the present study may not be discussed in an integral manner with other types of pathological gaits. Since regular walking already involves a standardized process that is expected by physicians and engineers responsible for developing algorithms to capture the most reliable biomechanical parameters from human locomotion, this process still requires more time to yield similar outcomes in pathological conditions. In this circumstance, our results may not be entirely representative of clinical populations, where gait patterns are more variable.

## 5. Conclusions

This study demonstrated that the OpenCap system produces strong agreement with most gait parameters analyzed during walking at various speeds when compared to MoCap. OpenCap had excellent agreement with MoCap for spatiotemporal gait variables across different walking speeds, with minimal mean biases and outstanding reproducibility. At the same time, from the joint angle standpoint, discrete joint ROM measures revealed greater variability, particularly at the hip and ankle, across all speeds. In contrast, continuous joint angle waveforms for the hip, knee, and ankle, as well as CoM displacement, displayed high consistency between systems across all tested speeds. Taken together, these findings highlight a nuanced profile for OpenCap. While it functions as a robust tool for spatiotemporal parameters and continuous kinematics, the identified limitations necessitate caution, especially in clinical settings with varying speeds. However, it is important to contextualize the magnitude of these errors. For the less reliable discrete ROM measures, the observed mean biases were within ranges often considered acceptable for clinical interpretation in gait analysis. Additionally, this systematic bias was smaller than the minimal detectable change (MDC) for these variables, indicating the system is sufficiently sensitive to track clinically significant progress time.

While the present validation was performed with a group of healthy participants, caution should be exercised when applying these findings to clinical populations. Pathological gait patterns often include asymmetrical movements, compensatory postures, and limited joint excursions, which could affect the accuracy of markerless motion capture. Future research should evaluate OpenCap’s performance in individuals with neurological or orthopedic impairments, such as those with post-stroke, Parkinson’s, or post-surgical gait, to determine if the good precision observed in healthy walking conditions also applies to pathological situations.

## Figures and Tables

**Table 1:** Mean and SD; Bias and its 95% Limits of Agreement (LoA); and agreement coefficients of gait parameters by speed: Intra-Class Correlation (ICC), Pearson’s correlation (r), and the minimal detectable change at the 95% confidence level (MDC).

Variable	Speed	MoCap Mean ± SD	OpenCap Mean ± SD	Bias (95% LoA)	ICC (2,1)	r	MDC
Stride Length (m)	Slow	1.286 ± 0.103	1.276 ± 0.102	−0.009 (−0.024, 0.006)	0.993	0.997	0.015
	Self	1.450 ± 0.062	1.439 ± 0.065	−0.012 (−0.030, 0.008)	0.969	0.988	0.019
	Fast	1.664 ± 0.082	1.652 ± 0.086	−0.010 (−0.037, 0.017)	0.984	0.992	0.027
Stride Time (s)	Slow	1.285 ± 0.113	1.283 ± 0.111	−0.002 (−0.012, 0.007)	0.999	0.999	0.009
	Self	1.116 ± 0.069	1.116 ± 0.068	0.000 (−0.005, 0.004)	0.999	0.999	0.004
	Fast	0.977 ± 0.056	0.976 ± 0.058	0.000 (−0.011, 0.011)	0.995	0.995	0.011
Double Support (s)	Slow	0.197 ± 0.036	0.190 ± 0.034	−0.007 (−0.022, 0.008)	0.950	0.973	0.015
	Self	0.149 ± 0.020	0.146 ± 0.018	−0.003 (−0.012, 0.006)	0.957	0.967	0.009
	Fast	0.111 ± 0.019	0.107 ± 0.020	−0.003 (−0.011, 0.005)	0.959	0.973	0.008
Cadence (Hz)	Slow	1.565 ± 0.137	1.568 ± 0.136	0.003 (−0.007, 0.012)	0.999	0.999	0.010
	Self	1.796 ± 0.112	1.797 ± 0.113	0.001 (−0.007, 0.009)	0.999	0.999	0.008
	Fast	2.052 ± 0.118	2.055 ± 0.120	0.000 (−0.024, 0.024)	0.995	0.995	0.024
Walking Speed (km.h^−1^)	Slow	3.614 ± 0.501	3.599 ± 0.505	−0.014 (−0.066, 0.037)	0.998	0.998	0.051
	Self	4.640 ± 0.373	4.616 ± 0.382	−0.022 (−0.082, 0.038)	0.995	0.997	0.060
	Fast	6.106 ± 0.478	6.059 ± 0.481	−0.044 (−0.135, 0.047)	0.992	0.995	0.091
Hip ROM (°)	Slow	41.875 ± 3.290	44.158 ± 3.183	2.318 (−1.427, 6.063)	0.665	0.829	3.745
	Self	45.456 ± 2.684	47.544 ± 3.155	2.031 (−0.960, 5.021)	0.709	0.898	2.990
	Fast	53.845 ± 3.093	55.505 ± 3.431	1.629 (−1.669, 4.926)	0.706	0.823	3.298
Knee ROM (°)	Slow	59.461 ± 4.594	59.079 ± 3.713	−0.389 (−7.010, 6.32)	0.647	0.650	6.711
	Self	60.408 ± 4.804	60.155 ± 4.846	0.412 (−5.549, 6.372)	0.776	0.770	5.960
	Fast	57.890 ± 4.742	57.939 ± 4.533	0.191 (−5.738, 6.120)	0.775	0.774	5.929
Ankle ROM (°)	Slow	21.712 ± 3.203	22.821 ± 3.116	1.164 (−4.038, 6.366)	0.591	0.622	5.202
	Self	21.004 ± 3.715	22.589 ± 3.815	1.313 (−3.550, 6.175)	0.700	0.739	4.862
	Fast	20.590 ± 3.006	22.408 ± 3.508	1.651 (−4.341, 7.643)	0.482	0.538	5.992

## Data Availability

The authors will provide the raw data supporting this article’s conclusions without unnecessary restriction.

## Abbreviations

The following abbreviations are used in this manuscript:

CoM	Center of Mass
G-G	Greenhouse–Geisser
ICC	Intraclass Correlation Coefficients
LoA	Limits of Agreement
MDC	Minimal Detectable Change
MoCap	Marker-based Motion Capture
.mot	Joint Angle Data
ROM	Range of Motion
RMSE	Root Mean Square Error
SPM	Statistical Parametric Mapping
TD	Touchdown
TO	Take-off
.trc	Three-dimensional Marker Position
